# Parenting and Healthy Teenage Lifestyles

**DOI:** 10.3390/ijerph17155428

**Published:** 2020-07-28

**Authors:** Paloma Alonso-Stuyck

**Affiliations:** Institute for Higher Family Studies, International University of Catalonia, 08017 Barcelona, Spain; palonsos@uic.es; Tel.: +34-676-686-641

**Keywords:** healthy lifestyles, parenting, adolescence behavioral autonomy, discrepancy between parents and teenage children

## Abstract

How can one promote adolescent adjustment toward a healthy lifestyle? The first step is to locate the healthy habit configuration within the family environment. The hypothesis is that, if adolescent lifestyles are assumed autonomously during adolescence, then it is very likely that they will last throughout life. How does this relate to parenting styles? After reviewing the literature of the last four decades on adolescent behavioral autonomy and scientific articles that link healthy lifestyles with parenting, several conclusions have been reached, such as the relevance of recovering the biopsychosocial richness of healthy lifestyles, the need to use a dialogue strategy to resolve discrepancies between adolescents and their parents, and the adequacy of the personalistic parenting style to promote adjusted adolescent behavioral autonomy, and with it maintain healthy lifestyles in the long term.

## 1. Introduction

The main objective of this article was to pinpoint which parenting styles promote adolescent adjustment according to recent research: What has been investigated so far and what aspects remain unknown? Why do certain healthy adolescent lifestyles not continue into adulthood? What changes occur if the way of dealing with discrepancies between parents and children regarding adolescent autonomy is modified? Before reviewing these variables throughout the last four decades and in order to interpret the results in greater depth, the following three constructs are presented: healthy lifestyles, parenting styles, and adolescent autonomy.

### 1.1. Healthy Lifestyles

This section exposes the need to restore the concept of healthy lifestyles that integrate the three personal dimensions: affective, conative, and cognitive, given that personal adjustment lies within the balance of those three domains. It is proposed that if childhood is understood as the period in which the affective relational style is configured, then it is during adolescence that the behavioral aspects of lifestyles are consolidated; and these lifestyles will continue into adulthood as long as they have been assumed autonomously.

To speak of healthy lifestyles, in their entirety, is to speak of personal adjustment. Most of the research on healthy lifestyles focuses on biorhythms—the physical dimensions of healthy habits—due to their connection with chronic diseases [1]. However, since 1948, the World Health Organization (WHO) has promoted a concept of health that also integrates psychological and social aspects, typical of the prevailing biopsychosocial paradigm in humanities and social sciences. In line with this concept of integration, it is specifically the three personal dimensions previously mentioned that are used most extensively in adolescent healthy lifestyle questionnaires [2,3,4] to evaluate personal adjustment.

**Figure 1:**
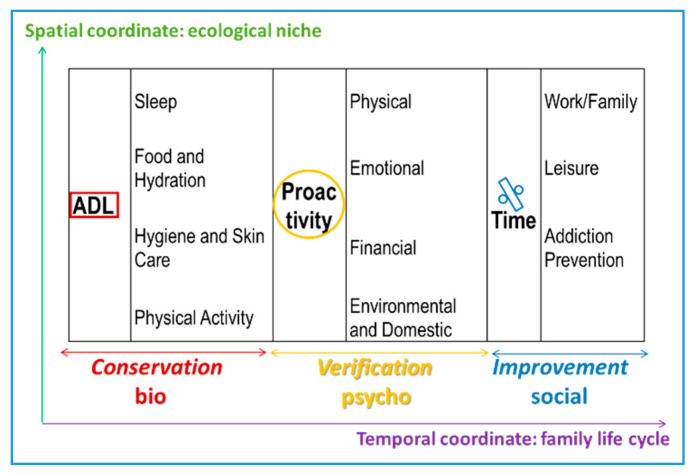
Biopsychosocial, personal, and motivational classification of healthy lifestyles.

In Figure 1, the healthy lifestyle behaviors and their connection to the personal dimensions and their motivations or basic tendencies are represented. The first category reflects the somatic habits: sleep, food and hydration, hygiene and skin care, and physical activity. These habits can be incorporated into the basic activities of daily living (ADL) [5]. The second category illustrates proactivity, a psychological attitude of initiative and responsibility toward one’s own physical, emotional, financial, and environmental and domestic health. In children and teenagers this attitude is associated with wealth and public safety [6]. The third category can be summarized as balance, the way in which one distributes one’s own time between work, family, and leisure. A proper balance between the personal, work, and social spheres translates into adequate time management, which may help prevent addiction problems. Meeting friends over the weekend for a few drinks is not the same as drinking alcohol every day, in the same way that spending half an hour a day doing sports is not the same as 4 hours every day, etc. Thus, the proportion of time devoted to a specific activity can reflect either a prevention factor or a risk factor in terms of addictions [7,8,9].

Figure 1 integrates the classical classification of the personal dimensions (affection, intelligence, and will) with basic human tendencies (conservation, verification, and improvement). The balance between these dimensions promotes personal adjustment and integrates all areas of the biopsychosocial paradigm. From the psychological point of view, the individual balance is rooted in the harmonic combination of the affective, cognitive, and conative dimensions, which are reciprocally rooted in the sensitivity, intelligence, and will that are reflected in the traits of self-transcendence: beauty, truth, good, and coexistence [10].

Therefore, when investigating healthy lifestyles, it becomes imperative to analyze cognitive and affective aspects and observable behaviors which can be collected through measurable data. To illustrate why this is necessary, we can use the following example: one cannot assume that a teenager who does not consume alcohol enjoys good psychosocial adjustment if that behavior does not correspond to an autonomous decision, or if the behavior is forced. This implies that, in addition to the observable result, it is convenient to analyze why a person acts in a specific way, what knowledge the action is based on, and how the person feels about that particular behavior. This would be the proper way to promote lifestyles that last throughout life because of their free nature.

It is precisely this balance between personal dimensions that is required to verify whether or not an act is truly human, free, because it implies consent and caveat [11]. In other words, the person understands the implications of what he/she decides to do and chooses to do it. Only from this internal governance that combines all three personal dimensions does a person act freely and have the ability to love.

**Figure 2:**
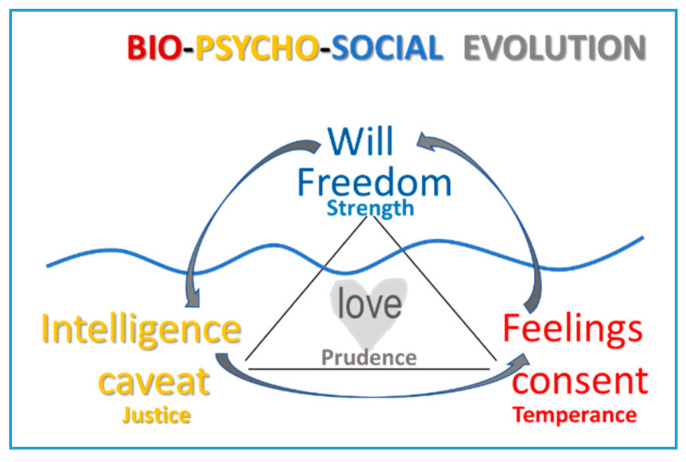
Dimensional analogy between health, personality, Aristotelian virtues, and Characteristics of free human action.

Figure 2 represents a truly free human act using the metaphor of the iceberg, with the wavy line representing the sea level. In this way, one can find that below the observable act dependent on will, intelligence and feelings are present: the teenager’s thoughts and feelings in regard to the act. In other words, the behavior’s emotional dimension has a somatic root—biological, sensitive, or sensory—that makes sense with the intervention of intelligence [12].

When a type of behavior is repeated, it becomes a stable trend, a lifestyle. These trends help us achieve the best versions of ourselves when we can balance what we feel, think, and want. This balance is known classically as virtue. In this way, their contributions are endorsed by evolutionary theories: a person’s developmental stage—sensorimotor, operational, and formal—of Piagetian theory is balanced by the Aristotelian virtues—temperance, strength, justice, and prudence. When adolescence is reached, a period begins in which the necessary developmental milestones have been achieved so that healthy lifestyles can be established, as shown in Figure 2.

Figure 2 illustrates the parallelism between the development of Aristotelian virtues, the health dimensions, and the personal dimensions. In fact, one might observe that positive psychology develops the classical cardinal virtues.

From this comparative analysis between personal dimensions, Aristotelian virtues, and biopsychosocial evolutionary theories, one can infer the importance of promoting a concept of healthy lifestyles that goes beyond basic biorhythms—a model that embraces the WHO’s proposal for individuals to assume the leading role in their health in all its biopsychosocial integrity. In addition to the biological prism of healthy lifestyles, the specific psychosocial aspects of proactivity, that is, initiative and purpose, can be found. This attitude favors establishing good intrapersonal and interpersonal relationships: with oneself and with others. It is a disposition that is exercised both in the private sphere—domestic, financial—and the community sphere—environmental. It configures the relational DNA, a personal healthy style, and a sustainable social style.

Neuroscience provides a definitive guideline to establishing long term healthy lifestyles by stating that when social pleasure guides education and work, the person interprets it as the highest form of learning [13]. In this sense, there is a consensus among psychological schools on the importance of early experiences on the child’s personality and therefore of the influence of the family environment in shaping this aspect of healthy lifestyles [14]. Personalistic psychology supports this statement by stressing that the person builds and grows in affectionate dialogue [15]. For children, an affectionate family environment provides a feeling of safety which allows them to develop the best personal versions of themselves.

In childhood, during the Piagetian sensorimotor period, the relational style of the individual is shaped by the type of attachment that is established during this period. This is known as attachment style which can be summarized as a mental outline of what interpersonal relationships are. This mental outline is configured during childhood and remains unconscious throughout the rest of one’s life. It is not based on logical decisions or reasoning but is an unconscious behavioral imprint. The way in which a child learns it is emotional and is driven by following and imitating significant people in their environment. In a similar way, this study’s approach is that in adolescence, the beginning of the formal thinking stage, the teenager’s lifestyle is configured and maintained if it is taken on autonomously [16]. It is therefore convenient to analyze the impacts of parenting styles on the acquisition of healthy lifestyles in adolescence. 

### 1.2. Parenting 

This second section includes the evolution of parenting, considering that healthy lifestyles—adolescent adjustment—are consolidated within the family environment. The importance of promoting adolescent autonomy emphasizing the three personal dimensions—affective, conative, and cognitive—is underlined.

**Figure 3:**
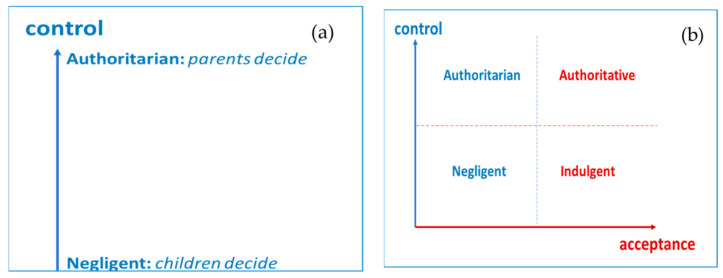
Evolution of the parenting educational style concept (**a**): one-dimensional; (**b**): two-dimensional.

There are numerous reviews [17,18,19,20,21] on the origins and evolution of parental educational styles. Figure 3a shows the simplest approach [22] that only addresses the type of control—conative dimension—while other models add the emotional dimension: responsiveness [23] Figure 3b Currently, the concept of parenting has become more complex and can be understood as a set of beliefs, feelings, and patterns of parental behavior that affect the psychosocial functioning of children [24]. Even though the authoritative style (high acceptance/implication and high coercion/imposition) was associated with adolescent adjustment at the beginning, nuances of this primacy soon emerged, according to the social context—horizontal vs. vertical, or individualistic vs. collectivist culture [25]. Currently, perhaps at the behest of globalization derived from the digital world, we have very novel results that indicate that the indulgent style (high acceptance/involvement) achieves a similar or greater adjustment than the authoritative style in different adolescent adjustment variables [26]. That was initially researched in Spain and Brazil [27,28], and lately it has been confirmed in other countries such as Germany and the United States [29,30,31], highlighting the importance of parental involvement in variables such as school adjustment or being a victim of cyberbullying [32,33,34]. 

**Figure 4:**
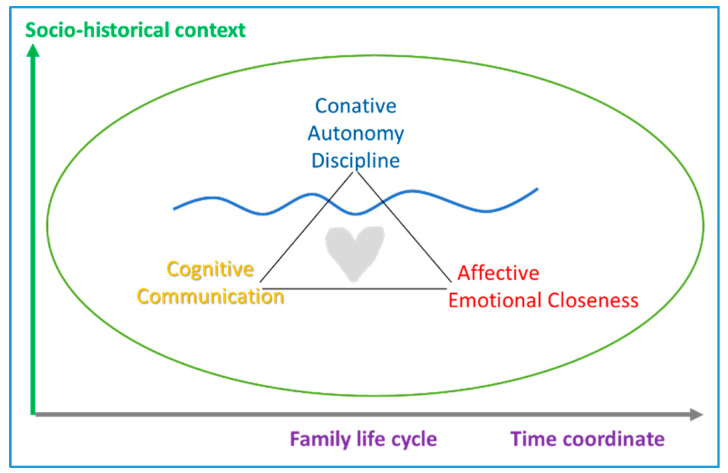
Personalistic parenting model [35].

Other studies speak of how to banish toxic family scenarios with compassion-centered parenting intervention programs [21], which add the cognitive—reflective—dimension of personality, in addition to the conative and affective ones contemplated in previous models. This is also the approach of the personalist model [35], which introduces a gradualness in the exercise of the three dimensions—conative, affective, and cognitive—as a way to match the different sociocultural scenarios and vital stages. Along with control and emotional openness—responsiveness—a communication style is considered that appeals to intelligence—cognitive dimension—to promote adolescent autonomy and make the internalization of healthy lifestyles in the long term possible, in line with the biopsychosocial, evolutionary, and contextual approach [36], as shown in Figure 4.

This parenting style is an introduction to the systemic approach, in which parenting styles are shaped by the contributions of parents and children alike, something that can be verified by the joint construction and family functioning models [19,37,38,39,40,41]. Through a proper human relationship—which integrates all the personal dimensions—the personal adjustment of both parents and children matures. When analyzing family interactions, psychoanalysis interprets behavioral problems as a failed process of individuation of adolescents from their parents [42]. Therefore, it is interesting to understand what parenting styles contribute to a healthy teenage autonomy that is able to sustain these healthy lifestyles previously acquired in the family in the long term.

### 1.3. Adolescence Autonomy

This third section highlights the type of autonomy that contributes to adolescent adjustment—autonomy that originates from the way in which parents and teenagers deal with the typical discrepancies of this period, which involves the three personal dimensions.

**Figure 5:**
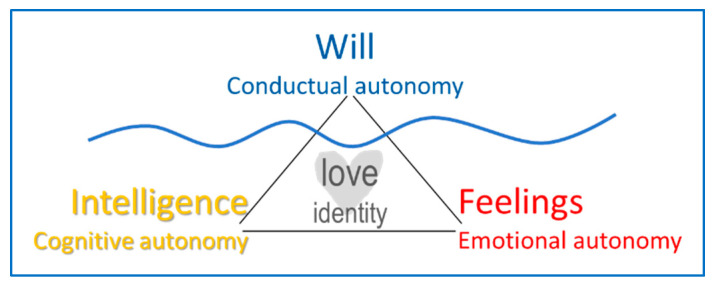
Contributions of the autonomy dimensions for healthy adolescent identity.

Given the social nature of the human being, healthy autonomy is placed in an intermediate state between independence and dependence, interdependence. It is a way of being and acting that permeates the whole person: the emotional, cognitive, and behavioral dimensions. Adolescents’ own evolutionary task [43], discovering and constructing their own identity, drives them to demand greater autonomy: they do not want to continue doing what their parents say but rather to start making their own decisions. This tends to generate discrepancies between parents and teenage children regarding the expectations as to *who is responsible for deciding what*. The way to deal with these discrepancies is closely related to the internalization of the decisions made [17,44]. If parents do not promote their children’s autonomy, children do not own their decisions and cannot take them on. On the contrary, if parenting styles support this evolutionary task, each of the dimensions of autonomy grows asynchronously, contributing to a healthy identity [45,46], as shown in Figure 5.

The type of confrontation has a big impact on healthy lifestyles in the short and long term. Therefore, it seems interesting to understand the beneficial impact of this asynchronous development of the different facets of autonomy and respect it [47,48]. It is also useful to understand how parenting styles contribute to the optimal resolution of the discrepancy between parents and adolescents. 

#### How to Deal with the Discrepancy between Parents and Children

**Figure 6:**
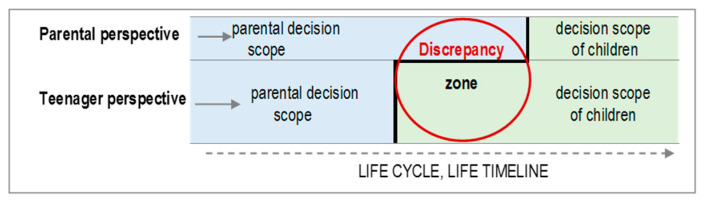
Delay phenomenon of parental expectations regarding adolescent autonomy.

The existence of the discrepancy between parents and adolescents regarding the expectations of decision-making is confirmed in the psychological evolutionary literature [49,50,51]. Despite the fact that it diminishes when the cultural gap between the two decreases [52], it is common for the amount of discretion expected by adolescents to be greater than that estimated by their parents, as shown by the delay phenomenon in Figure 6.

The way in which possible discrepancies between adolescents and their parents are dealt with seems to be decisive for adolescents making their own decisions. It is not so much about who is right, and not even about what behaviors are included in the decision areas: moral, socio-conventional, and private [17,53]; but rather the way in which these discrepancies are solved. These coping strategies have been classified, leaving the frequency as follows: 20% negotiation, 30% submission of the children to the opinion of the parents, and 50% withdrawal or change of focus of the discussion [17,54]. 

The resolution of this discrepancy results in consequences in all areas of adolescent adjustment, academic achievement [55,56], conduct disorders [26,57] beginning as dependencies, internalized and externalized symptoms of mental health [38,58,59,60,61,62], etc.

The 20% referring to the *negotiating parents* seem to respond to the personalistic parenting style, to a communication style that favors autonomy, adapting to the characteristics of the children and the sociocultural context. To encourage this negotiating stance, parents will find it helpful to consider that the demand for autonomy is an adolescent rite [63]; that children need your support to maintain their motivation [64] and self-esteem [65]; and to manage the insecurity of liquid society [66].

It can therefore be concluded that family counseling programs should promote a parenting style that caters to the three personal dimensions of parents and children. There should especially be a focus on the optimal way of facing arguments with adolescents, so that they accept the decisions that are being, made and with them, the healthy family lifestyles.

In the following sections, the analysis of the relationship between the three variables (healthy lifestyles, parenting, and adolescent autonomy) will be presented.

## 2. Method

**Figure 7:**
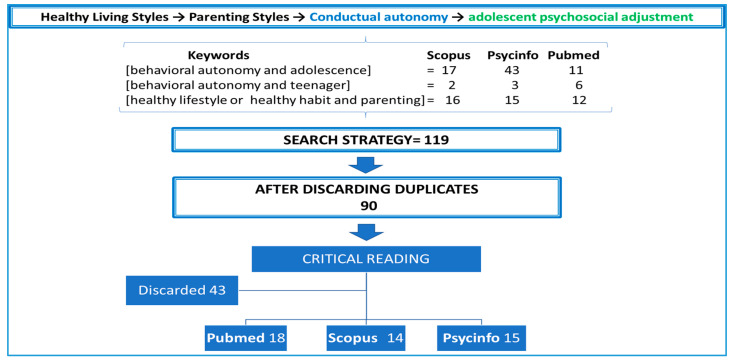
Bibliographic search algorithm.

A bibliographic search was carried out using three databases. Scopus was chosen in first place because it is one of the most prestigious international databases, despite its multidisciplinary nature. Psycinfo was the second database chosen due to the psychological approach of this study. The last database to be selected was Pubmed, in which the largest number of quality articles on different aspects of healthy lifestyles was expected. For the search strategy, the same keywords were used in all databases—behavioral autonomy, adolescence, teenager, healthy lifestyle, and healthy habits—thereby obtaining different results, as expected. The strategy is shown in Figure 7. The number of articles obtained with the key phrase healthy lifestyle was substantially reduced when crosschecking them with the keyword Parenting.

The 47 publications reviewed were as follows: “acta psychologica sinica,” “adolescence,” “adolescent psychiatry: developmental and clinical studies,” “aggressive behavior,” “annales médico-psychologiques,” “annals of behavioral medicine: a publication of the society of behavioral medicine,” “asia pacific journal of counselling and psychotherapy,” “australian journal of educational and developmental psychology,” “behavior therapy,” “behavioral psychology / psicología conductual: revista internacional clínica y de la salud,” “british journal of educational psychology,” “ca: a cancer journal for clinicians,” “cahiers critiques de thérapie familiale et de pratiques de réseaux,” “clinical child and family psychology review,” “clinical journal of pain,” “çocuk ve gençlik ruh sağliği dergisi,” “child development,” “development and psychopathology,” “developmental science,” “early human development,” “ethnic and racial studies,” “european psychologist,” “family and community health: the journal of health promotion and maintenance,” “frontiers in psychology,” “giornale di neuropsichiatria dell’età evolutiva,” “health education,” “infancia y aprendizaje: journal for the study of education and development,” “infanzia e adolescenza,” “international journal of adolescence and youth,” “international journal of behavioral development,” “journal of addictions nursing,” “journal of adolescence,” “journal of adolescent research,” “journal of behavioral medicine,” “journal of consulting and clinical psychology,” “journal of child and family studies,” “journal of family psychology: jfp: journal of the division of family psychology of the American psychological association (division 43),” “journal of interpersonal violence,” “journal of pediatric psychology,” “journal of research on adolescence,” “journal of substance abuse treatment,” “journal of youth and adolescence,” “kindheit und entwicklung: zeitschrift für klinische kinderpsychologie,” “motivation and emotion,” “neuroethics,” “neuropsychiatrie de l’enfance et de l’adolescence,” “new directions for child and adolescent development,” “perspectives on psychological science: a journal of the association for psychological science,” “psichologija,” “psychiatria hungarica,” “revista iberoamericana de diagnóstico y evaluación psicologica,” “social psychology of education: an international journal,” “the american psychologist,” “turk psikoloji dergisi,” “vulnerable children and youth studies,” “youth and society.”

Once the information was read and systematized, the analysis categories were used to delve into the associations between the descriptors or keywords. In the qualitative analysis, a total of 47 articles were reviewed. In terms of the aim of this study, to pinpoint what type of parenting styles promote adolescent adjustment according to recent research: What has been investigated so far and what aspects remain unknown? Why do certain healthy adolescent lifestyles not continue into adulthood? What change occurs if the way of dealing with discrepancies between parents and children regarding adolescent autonomy is modified? The questions were used as a guide for the analysis of the articles which allowed the identification of relevant aspects.

## 3. Development and Discussion

A total of 119 articles were identified. Following a review for duplicates, the number of articles was reduced to 90 and following a critical reading the number was even further reduced to 47. They are cited in the discussion and conclusions sections according to the color of the database (Scopus or Psycinfo). Pubmed articles have not been cited in the discussion because they refer to lifestyle aspects related to the physical dimension, whereas this study focused on the psychosocial aspects of adolescent adjustment.

Based on the questions posed for this article, five analysis categories stood out: (1) healthy behavioral autonomy as a protective factor for adolescent lifestyles, (2) the meaning of healthy adolescent autonomy, (3) parenting styles that promote healthy adolescent autonomy, (4) how to solve the discrepancy between parents and teenage children, and (5) the interactive nature of the conflict resolution process.

Firstly, some articles reinforce the main hypothesis, highlighting healthy behavioral autonomy as a protective factor against teenage risk behaviors [67], conduct disorders [68,69], and socio-emotional problems [70]. A possible explanation for this is that if the main evolutionary task of teenagers is to discover their own identity [43], if they feel that they take the lead of their behavior, they will not need to look for alternative environments in which to prove themselves. Sometimes the adjustment comes as a consequence of promoting school adjustment [71]. It could be because if your fellow students recognize your worth, you do not need to look for it in other groups outside of school [55,56].

The importance of timing is highlighted, given that behavioral autonomy in early adolescence is associated with crime [68,72], and with adolescent adjustment in the later stages. This confirms that prudent decisions are acquired gradually, at first on accidental issues and little by little on issues of greater relevance [17,53]. It also confirms that the three personal dimensions grow at different rates: they need time to integrate into coherent and healthy behavior [45,46].

In regard to the meaning of healthy adolescent autonomy, behaviors are addressed for which adolescents are considered autonomous [73] with parents being the main reference when making decisions, especially in matters of moral significance [74]. This means that, as indicated by the attachment theory, the bond with the parents does not need to break [75], something that has been studied in diverse cultures—[76] France, [77] Milan, and [78] Istanbul. In addition, closeness to the parent delays the appearance of antisocial disorders [79]. All this highlights the importance of an adequate gradation between the emotional, behavioral, and cognitive dimensions of autonomy, as proposed by the autonomy typology [48].

Thus, an attempt was made to define what is meant by healthy adolescent behavioral autonomy [80], studying its connection with self-esteem [67] and with different patterns of individuation [81]. A lack of healthy behavioral autonomy as a possible physical origin is also suggested in premature infants, due to a deficit in executive functions [79]. In each culture, the content and process of adolescent behavioral autonomy will acquire its own nuances. However, the attachment style seems decisive for adolescent adjustment. Secure attachment seems to be associated with healthy emotional and behavioral autonomy.

Furthermore, healthy adolescent autonomy can be considered a prevention factor for substance dependence [42], specifically cannabis [82], for the recurrence of depression symptoms [83,84], and in general for externalizing symptoms of mental disorders, as shown by the recent review [85]. It is also associated with other aspects of physical health, such as headache [86], adherence to treatment in patients with diabetes [87] or their resilient attitude toward health [88], and in general, commitment to the treatment of chronic diseases [84]. These data reflect the difference between acting on extrinsic or intrinsic motivation. Behavior that is assumed in a personal way, through intrinsic motivation, manifests itself in all personal situations, regardless of the obstacles, and with behavioral autonomy.

The way in which adolescents achieve this type of healthy autonomy is linked to the parenting style, to the family environment [89]. For teenagers to sense a positive environment, it is imperative that time be spent together. In this sense, healthy autonomy can be associated with the parents’ schedule [90]. This influence of parenting on adolescent adjustment gradually decreases throughout early, mid, and late adolescence [69]. 

The indulgent style is associated with healthier adolescent autonomy in some countries [91], as indicated in the introduction, with adolescents raised in institutions achieving less autonomy than those who live with family [92]. From these results one can interpret that the development of behavioral autonomy is based on the safety provided by the parents, in the accepting way in which parents embrace and reason with their children regarding decisions. This feeling of belonging, of feeling loved, is also associated with adolescent self-esteem and adjustment.

As part of the parenting style, the way to solve the discrepancy between parents and children regarding decision-making is especially linked to adolescent adjustment. To explain this, the theory of *structural analysis of social behavior*—SASB—[93] is used, considering autonomy as a teenager’s rite of passage [94], an achievement for which one must engage in negotiation [95]. These results are in line with the Piagetian theory, according to which, if there is no argument, ideas are not owned, behaviors are not assimilated. The evolutionary theories presented in the introduction corroborate these results. Arguing can be considered a teenage rite of passage, somewhat normative; but passive resolution of these arguments, without disagreement, without offering resistance or dialogue, will not allow for internalization, as exposed by the Piagetian theory, and therefore, the adopted lifestyles will not be maintained over time, as they have not been taken on at a personal level.

The magnitude of the discrepancy depends on cultural beliefs, as indicated by the Social Dominance Theory [87], being greater in urban than in rural areas [59], and greater in immigrant minorities—in Europe and America—than in the families of the country [96,97]. These results show the influence of the environment. They may also reflect a way of resolving arguments in more traditional, non-negotiating settings [17,54].

In regard to the process of solving the conflict between parents and adolescents, the interactive nature of the process stands out; in fact, it is said that it is a catalyst for the transformation of family relationships [98] and that its resolution improves with a collaborative environment between parents and children [93]. This does not mean that solving the conflict falls entirely on parents allowing children to present their arguments, as it is also the children’s task to listen to their parent’s reasons.

Examples of the reciprocity of the conflict resolution process can be seen in the following examples: to reduce symptoms of externalization, the same efficacy of results can be obtained either through parental education or adolescent therapy [99]; to reduce the climate of hostility in the family, one solution is to educate parents in self-regulation and social cognition [85], something that was predicted by the General Theory of Crime and Delinquency [70]. The quality of the relationship between parents and children depends on both.

Once the results of the analysis are grouped into these five categories, one line of reasoning appears that can unite all of them together. This line of reasoning is described next, and a mental map is developed, a map that will guide the conclusion.

### Development of a Mental Map and Critical Argumentation of the Results

Some constants were extracted from the analysis of the articles of the last four decades (1979–2000). Firstly, given the global nature of the review—different cultural settings—the influence of the parenting style on adolescent healthy autonomy, the meaning of this, and its relationship with a broader, biopsychosocial conception of healthy lifestyles acquires different nuances according to the time-space coordinates.

- From a *spatial* point of view, parenting guidelines must be adapted to geographic contexts, and even in the same country, to urban or rural settings. At a *micro* level, one can observe that the unit of analysis of the relational environment has expanded, from a unidirectional approach focused on the behavior of adolescents to a systemic approach attentive to the relational quality between parents and children—a quality that requires family interaction, which affects not only the behavioral dimension, but also the affective and cognitive personality.
- Regarding the *temporal* coordinate, timing stands out on the one hand, and on the other hand there is the appropriate combination—asynchronous—of the facets of autonomy—behavioral, cognitive, or emotional—in each adolescent stage: early, mid, and late. [63].

Moreover, the persistence of the delay, the gap between the desire for adolescent autonomy and the areas of freedom given by the parents, is established. That is why the strategies for solving these discrepancies become especially relevant strategies that include the involvement of all the personal dimensions of parents and children.

In addition, adolescent autonomy is linked to the variables already mentioned in the introduction by exposing the state of the art—academic achievement, conduct disorders, dependencies, externalizing and internalizing symptoms of mental health disorders, adherence to the treatment of chronic diseases, and acquiring physical activity habits [64].

**Figure 8:**
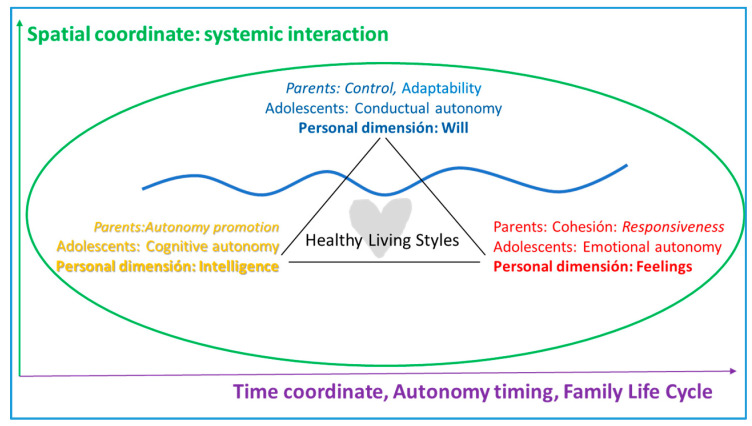
Mind map and integration of results.

Placing the obtained conclusions in the personality dimensions allows them to be integrated. In this way, applying them in prevention, counseling, and family therapy programs, in order to promote healthy lifestyles becomes more manageable. Figure 8 shows this integration:

## 4. Conclusions 

The theoretical review carried out in this study allowed the identification of five analysis categories that help understand this particular issue.

(1) In the family lifecycle, if childhood is presented as the sensitive period to establish the relational style according to the type of attachment [65], adolescence—with the debut of formal thought and the desire for autonomy—emerges as a critical stage for the consolidation of behavioral autonomy and therefore of healthy lifestyles in the short and long term.
(2) This comprehensive concept of a healthy lifestyle refers to the balance between the personal dimensions—affective, cognitive, and conative—and can be understood as personal adjustment. Therefore, adolescent adjustment translates into behavioral autonomy and a healthy lifestyle.
(3) Given the connection of healthy lifestyles and parenting, it is advisable to sensitize families in regard to the personal and economic benefits of addressing healthy lifestyles in all its biopsychosocial integrity, not limited to biorhythms, but also paying attention to the psychological and social aspects related to time management and attitude towards activities and relationships.
(4) During this developmental stage, the way in which parents and children resolve the discrepancy regarding the areas of decision that the adolescent yearns for is decisive for teenage children to embrace family life habits as their own, so that they can internalize healthy lifestyles and with them promote adolescent adjustment.
(5) The personalistic parenting style, being based on the personal dimensions—just like the healthy lifestyles—can easily be integrated and translated into family intervention and counseling programs. The indication is clear: to respect the interaction of the process, the involvement of parents and children, thereby avoiding a one-way approach as far as possible.

### Limitations and Outlook

Despite the theoretical and methodological development of positive psychology in relation to the psychological factors of healthy lifestyles, some concepts require more research in order to be evaluated with greater reliability, such as the proactive attitude towards physical, emotional, financial, and environmental health, etc.

A traditional objective trend in educational psychology is to guide, so that extrinsic motivation gradually gives way to intrinsic and transcendent motivation. A possible line of research would be to transfer this challenge to family counseling and intervention programs, showing the close connection between promoting adolescent autonomy and intrinsic motivation.

To navigate the uncertain waves of a liquid society, many families turn to family counseling services while looking for guidelines to help educate their children, especially during the adolescent stage. The personalistic parenting style is characterized by adapting the dimensions of control, acceptance, and communication to each unrepeatable situation, deepening the perceptions of parents and teenage children in terms of who should make decisions regarding moral, socio-conventional, and private issues. It also implies designing a methodology of adjustment to bring the expectations of both parents and children closer and is therefore presented as a useful line of research to promote healthy lifestyles.

Currently, an essential characteristic of adolescent lifestyles, which some authors call state inconsistency [66], is being adopted by many adults who see their autonomy reduced due to job instability.

## References

[1] Di Luzio S.S., Procentese F., Guillet-Descas E. (2019). Physical Activity in Adolescence and Substance Use: Factors of Interdependence between Local Community and Relational Micro-Systems. J. Child Adolesc. Subst. Abuse.

[2] Chen M.Y., Yang R.J., Liou Y.M. (2003). Adolescent health promotion scale: Development and psychometric testing. Public Health Nurs..

[3] Gillis A.J. (1997). The Adolescent Lifestyle Questionnaire: Development and psychometric testing. Can. J. Nurs. Res..

[4] Hendricks C., Murdaugh C., Pender N. (2006). The Adolescent Lifestyle Profile: Development and psychometric characteristics. J. Natl. Black Nurses Assoc..

[5] WHO. https://www.who.int/classifications/icf/en/.

[6] Moffitt T.E., Arseneault L., Belsky D., Dickson N., Hancox R.J., Harrington H., Caspi A. (2011). A gradient of child self-control predicts health, wealth, and public safety. Proc. Natl. Acad. Sci. USA.

[7] Riquelme M., García O.F., Serra E. (2018). Psychosocial mismatch in adolescence: Parental socialization, self-esteem and substance use. An. Psicol..

[8] Ohannessian C. (2012). Parental Problem Drinking and Adolescent Psychosocial Adjustment: The Mediating Role of Adolescent-Parent Communication. J. Adolesc. Res..

[9] Taylor R., Redding S., Murphy M., Sheley P. (2011). Association of poverty with family relations and children’s and adolescents’ socioemotional adjustment. Handbook on Family and Community Engagement.

[10] Sánchez A. (2010). Hope and Coexistence in Leonardo Polo’s Philosophy. Mayéutica.

[11] Pardo A. (2008). About the human act. Pers. Bioét..

[12] Bisquerra R. (2011). Emotional Education. Proposals for Educators and Families.

[13] Bueno D. (2017). Neuroscience for Educators.

[14] Redding S., Murphy M., Sheley P. (2011). Handbook on Family and Community Engagement.

[15] Buber M. (1993). Me and You.

[16] Oliva A., Parra A., Arranz E. (2008). Relational parenting styles and adolescent adjustment. Infanc y Aprendiz J. Stud. Educ. Dev..

[17] Alonso-Stuyck P. (2005). Discrepancy between Parents and Children in the Perception of Family Functioning and Development of Adolescent Autonomy. Ph.D. Thesis.

[18] Torio S., Peña J.V., Rodríguez M.C. (2008). Parenting educational styles. Bibliographic review and theoretical reformulation. Educ Theory.

[19] Aroca C., Cánovas P. (2012). The parenting educational styles from the interactive and joint construction models. Educ. Theory.

[20] Jorge E., González C. (2017). Parenting Styles: A Theoretical Review. Psychol. Rep..

[21] Kirby J.N. (2020). Nurturing Family Environments for Children: Compassion-Focused Parenting as a Form of Parenting Intervention. Sci. Educ..

[22] Baumrind D. (1991). Parenting Styles and Adolescents.

[23] Maccoby E.E., Martin J.A., Hetherington E.M., Mussen P.H. (1983). Socialization in the context of the family: Parent-child interaction. Handbook of Child Psychology: Socialization, Personality and Social Development.

[24] Perales M.J., Bisquert M., Sahuquillo P. Definition of the construct of family educational styles for the EVALEF questionnaire. Proceedings of the ACTS IV International Congress.

[25] Soenens B., Park S.-Y., Mabbe E., Vansteenkiste M., Chen B., Van Petegem S., Brenning K. (2018). The Moderating Role of Vertical Collectivism in South-Korean Adolescents’ Perceptions of and Responses to Autonomy-Supportive and Controlling Parenting. Front. Psychol..

[26] Aymerich M., Musitu G., Palmero F. (2018). Family Socialization Styles and Hostility in the Adolescent Population. Sustainability.

[27] Martínez I., García J.F. (2007). Impact of parenting styles on adolescents’ self-esteem and internalization of values in Spain. Span. J. Psychol..

[28] Martínez I., García J.F. (2008). Internalization of values and self-esteem among Brazilian teenagers from authoritative, indulgent, authoritarian, and neglectful homes. Adolescence.

[29] Martínez I., García F., Fuentes M.C., Veiga F., García O.F., Rodrigues Y., Serra E. (2019). Researching parental socialization styles across three cultural contexts: Scale ESPA29 bi-dimensional validity in Spain, Portugal, and Brazil. Int. J. Environ. Res. Public Health.

[30] García F., Serra E., Garcia O.F., Martinez I., Cruise E. (2019). A third emerging stage for the current digital society? Optimal parenting styles in Spain, the United States, Germany, and Brazil. Int. J. Environ. Res. Public Health.

[31] Martínez I., Cruise E., García Ó.F., Murgui S. (2017). English validation of the parental socialization scale—ESPA29. Front. Psychol..

[32] Katz I., Lemish D., Cohen R., Arden A. (2019). When parents are inconsistent: Parenting style and adolescents’ involvement in cyberbullying. J. Adolesc..

[33] Serna C., Martínez I. (2019). Parental Involvement as a Protective Factor in School Adjustment among Retained and Promoted Secondary Students. Sustainability.

[34] Martínez I., Murgui S., García O.F., García F. (2019). Parenting in the digital era: Protective and risk parenting styles for traditional bullying and cyberbullying victimization. Comput. Hum. Behav..

[35] Alonso-Stuyck P. (2019). Which Parenting Style Encourages Healthy Lifestyles in Teenage Children? Proposal for a Model of Integrative Parenting Styles. Int. J. Environ. Res. Public Health.

[36] Gilbert P. (2019). Psychotherapy for the 21st century: An integrative, evolutionary, contextual biopsychosocial approach. Psychol. Psychother. Theory Res. Pract..

[37] Olson D.H. (2000). Circumplex Model of Marital and Family Systems. J. Fam. Ther..

[38] Yeung W.K., Tsang E., Chen H. (2019). Parental Socialization and Development of Chinese Youths: A Multivariate and Comparative Approach. Int. J. Environ. Res. Public Health.

[39] Shimotomai A. (2020). Parental social power, co-parenting, and child attachment: Early to late Japanese adolescence transitions. Curr. Psychol..

[40] Camacho P., León C., Silva I. (2009). Family functioning according to Olson’s circumplex model in adolescents. Herediana Nurs. Mag..

[41] Biglan A., Flay B.R., Embry D.D., Sandler I.N. (2012). The critical role of nurturing environments for promoting human well-being. Am. Psychol..

[42] Brouard M., Johnston G. (2009). “Chemical romance” or problems of separation: Ecstasy consumption during adolescence. Neuropsychiatr. Enfance Adolesc..

[43] Erikson E. (1985). El Ciclo Vital Completado.

[44] Bi X., Yang Y., Li H., Wang M., Zhang W., Deater-Deckard K. (2018). Parenting styles and parent–adolescent relationships: The mediating roles of behavioral autonomy and parental authority. Front. Psycholog..

[45] Hancock D. (2014). Consequences of Parenting on Adolescent Outcomes. Societies.

[46] Gómez E. (2008). Adolescence and family: Review of the relationship and communication as risk or protection factors. IJPES.

[47] Ryan R.M., Lynch J.H. (1989). Emotional Autonomy versus Detachment: Revisiting the Vicissitudes of Adolescence and Young Adulthood. Child. Dev..

[48] Alonso-Stuyck P., Zacarés J.J., Ferreres A. (2018). Emotional separation, autonomy in decision-making, and adjustment in adolescence: A proposed typology. J. Child. Fam. Stud..

[49] Rappoport L. (1986). Personality from 13 to 15 Years Old.

[50] Steinberg L., Morris A.S. (2001). Adolescent development. Annu. Rev. Psychol..

[51] De los Reyes A., Ohannessian C. (2019). Discrepancies between Adolescent and Parent Reports about Family Relationships. Child. Dev. Perspect..

[52] Wang-Schweig M., Miller B.A. (2019). Correction to: Examining the Interdependence of Parent–adolescent Acculturation Gaps on Acculturation-based Conflict: Using the Actor-Partner Interdependence Model. J. Youth Adolesc..

[53] Rote W.M., Smetana J.G. (2016). Patterns and predictors of mother–adolescent discrepancies across family constructs. J. Youth Adolesc..

[54] Zimmer-Gembeck M.J., Collins W.A., Adams G.R., Berzonsky M. (2003). Autonomy development during adolescence. Blackwell Handbook of Adolescence.

[55] Pallock L., Lamborn S.D. (2006). Beyond parenting practices: Extended kinship support and the academic adjustment of African-American and European-American teens. J. Adolesc..

[56] Lamborn S., Nguyen D.T. (2004). Educational Psychology, University of Wisconsin, Milwaukee, WI, USAfrican American Adolescents’ Perceptions of Family Interactions: Kinship Support, Parent-Child Relationships, and Teen Adjustment. J. Youth Adolesc..

[57] Özdemir Y., Vazsonyi A.T., Çok F. (2017). Parenting processes, self-esteem and aggression: A mediation model. Eur. J. Dev. Psychol..

[58] Solís G.L., Manzanares E. (2019). Parental Psychological Control and Internalized and Externalized Problems of Adolescents in Metropolitan. Rev. Colomb. Psicolog..

[59] Gonzales-Backen M. (2019). Parenting practices and adjustment profiles among Latino youth in rural areas of the United States. Soc. Sci.

[60] Xu J., Ni S., Ran M., Zhang C. (2017). The Relationship between Parenting Styles and Adolescents’ Social Anxiety in Migrant Families: A Study in Guangdong, China. Front. Psychol..

[61] Savard A., Joussemet M., Emond Pelletier J. (2013). The benefits of autonomy support for adolescents with severe emotional and behavioral problems. Motiv. Emot..

[62] Spirito A., Hernández L., Cancilliere M.K., Graves H., Barnett N. (2015). Improving Parenting and Parent-Adolescent Communication to Delay or Prevent the Onset of Alcohol and Drug Use in Young Adolescents with Emotional/Behavioral Disorders: A Pilot Trial. J. Child. Adolesc. Subst..

[63] Alonso-Stuyck P., Aliaga F. (2017). Demand for autonomy in the relationship between adolescents and their parents. Estud. Sobre Educ..

[64] Gillison F., Sebire S., Standage M. (2012). What motivates girls to take up exercise during adolescence? Learning from those who succeed. Br. J. Health Psychol..

[65] Oliva A. (2011). Attachment in adolescence. Acción Psicológica.

[66] Hurrelmann K., Quenzel G. (2015). Lost in transition: Status insecurity and inconsistency as hallmarks of modern adolescence. Int. J. Adolesc. Youth.

[67] Marsh P., McFarland F.C., Allen J.P., Boykin M.K., Land D. (2003). Attachment, autonomy, and multifinality in adolescent internalizing and risky behavioral symptoms. Dev. Psychopathol..

[68] Haase C.M., Tomasik M.J., Silbereisen R.K. (2008). Premature behavioral autonomy: Correlates in late adolescence and young adulthood. Eur. Psychol..

[69] Studsrød I., Bru E. (2009). The role of perceived parental socialization practices in school adjustment among Norwegian upper secondary school students. Br. J. Educ. Psychol..

[70] Pinquart M. (2018). Parenting as protective factor against aggressive and oppositional behavior of children? Stock taking and. Kindheit und Entwicklung Zeitschrift für Klin Kinderpsychologie.

[71] Álvarez-García D., García T., Barreiro-Collazo A., Dobarro A., Antúnez Á. (2016). Parenting style dimensions as predictors of adolescent antisocial behavior. Front. Psychol..

[72] Brauer J.R. (2017). Cultivating conformists or raising rebels? Connecting parental control and autonomy support to adolescent delinquency. J. Res. Adolesc..

[73] Sigafoos A.D., Feinstein C.B., Damond M., Reiss D., Feinstein S.C., Esman A.H. (1988). The measurement of behavioral autonomy in adolescence: The Autonomous Functioning Checklist. Developmental and Clinical Studies.

[74] Young J.W., Ferguson L.R. (1979). Developmental changes through adolescence in the spontaneous nomination of reference groups as a function of decision content. J. Youth Adolesc..

[75] Carreras M.A., Brizzio A., González R., Mele S., Casullo M.M. (2008). Attachment styles in romantic and non-romantic bonds. Comparative study with Argentine and Spanish young people. Rev. Iberoam Diagnóstico Evaluación Psicol..

[76] Delage M. (2008). Attachment and adolescence. Treatment implications. Cah. Crit. Thérapie Fam. Prat Réseaux.

[77] Costantino E., Limonta A., Maggiolini A., Crugnola C.R. (2011). Attachment, emotional and behavioral problems in adolescence. Infanz Adolesc..

[78] Akyüz D., Şendil G. (2014). The assessment of the mediating role of autonomy in the relationship between attachment s. Çocuk Ve Gençlik Ruh Sağlığı Dergisi.

[79] Mata A.D., van Dulmen M.H. (2012). Adult-onset antisocial behavior trajectories: Associations with adolescent family processes and emerging adulthood functioning. J. Interpers. Violence.

[80] Smetana J.G., Campione-Barr N., Daddis C. (2004). Longitudinal development of family decision making: Defining healthy behavioral autonomy for middle-class African American adolescents. Child. Dev..

[81] Levpušček M.P., Gril A. (2010). Patterns of individuation in Slovenian adolescents and their relationship with adolescents’ perceptions of parents, friends and teachers. Behav. Psychol./Psicol. Conduct. Rev. Int. Clín. Salud.

[82] Laguerre C.E., Vavassori D., Fernandez L. (2015). Parental contributions and separation anxiety on adolescents’ cannabis use. J. Addict. Nurs..

[83] Eagleton S.G., Williams A.L., Merten M.J. (2016). Perceived behavioral autonomy and trajectories of depressive symptoms from adolescence to adulthood. J. Child. Fam. Stud..

[84] Polizzi S., Polizzi M., Zazzaro C., Raia V. (2017). Anxious-drepressed traits valutation in pediatric cystic patients and in their parents: A study. Giornale di Neuropsichiatria dell’Età Evolutiva.

[85] Marusak H.A., Thomason M.E., Sala-Hamrick K., Crespo L., Rabinak C.A. (2018). What’s parenting got to do with it: Emotional autonomy and brain and behavioral responses to emotional conflict in children and adolescents. Dev. Sci..

[86] Palermo T.M., Putnam J., Armstrong G., Daily S. (2007). Adolescent autonomy and family functioning are associated with headache-related disability. Clin J. Pain.

[87] Drotar D., Ittenbach R., Rohan J.M., Gupta R., Pendley J.S., Delamater A. (2013). Diabetes management and glycemic control in youth with type 1 diabetes: Test of a predictive model. J. Behav. Med..

[88] Rohan J.M., Huang B., Pendley J.S., Delamater A., Dolan L., Reeves G., Drotar D. (2015). Predicting health resilience in pediatric type 1 diabetes: A test of the resilience model framework. J. Pediatr. Psychol..

[89] Parra Á., Oliva A. (2006). Relevant dimensions of parenting style during adolescence: A longitudinal study. Infancy Aprendiz J. Stud. Edururalc Dev..

[90] Feldman S.S., Wood D.N. (1994). Parents’ expectations for preadolescent sons’ behavioral autonomy: A longitudinal study of correlates and outcomes. J. Res. Adolesc..

[91] Musaaǧaoǧlu C., Güre A. (2005). Ergenlerde davranişsal özerklik ile algilanan ana-baba tutumlari arasindaki ïlişkiler. Turk. Psikol. Derg..

[92] Tulviste T. (2011). Autonomy, educational plans, and self-esteem in institution-reared and home-reared teenagers in Estonia. Youth Soc..

[93] Beveridge R.M., Berg C.A. (2007). Parent-adolescent collaboration: An interpersonal model for understanding optimal interactions. Clin. Child. Fam. Psychol. Rev..

[94] Stanwyck D.J. (1983). Self-esteem through the life span. Fam. Community Heal. J. Heal. Promot. Maint..

[95] Fleming M. (2005). Adolescent autonomy: Desire, achievement and disobeying parents between early and late adolescence. Aust. J. Educ. Dev. Psychol..

[96] Huiberts A., Oosterwegel A., VanderValk I., Vollebergh W., Meeus W. (2006). Connectedness with parents and behavioural autonomy among Dutch and Moroccan adolescents. Ethn. Racial Stud..

[97] Roche K.M., Caughy M.O., Schuster M.A., Bogart L.M., Dittus P.J., Franzini L. (2014). Cultural Orientations, Parental Beliefs and Practices, and Latino Adolescents’ Autonomy and Independence. J. Youth Adolesc..

[98] Collins W.A., Laursen B., Mortensen N., Luebker C., Ferreira M. (1997). Conflict processes and transitions in parent and peer relationships: Implications for autonomy and regulation. J. Adolesc. Res..

[99] Sturge-Apple M.L., Li Z., Martin M.J., Jones-Gordils H.R., Davies P.T. (2019). Mothers’ and fathers’ self-regulation capacity, dysfunctional attributions and hostile parenting during early adolescence: A process-oriented approach. Dev. Psychopathol..

